# The Impact of Inoculation Rate and Order on Physicochemical, Microstructural and Sensory Attributes of Probiotic Doogh 

**Published:** 2013

**Authors:** Said Reza Rahmdar, Mohammad Rahmati Roudsari, Ahmad Javanmard, Amir Mohammad Mortazavian, Sara Sohrabvandi

**Affiliations:** a *Shahid Beheshti University of Medical Sciences, P.O. Box 19395-4741, Tehran, Iran. *; b *Skin Research Center, Vice Presidency of Research and Technology, Shahid Beheshti University of Medical Sciences, P.O. Box 19395-4741, Tehran, Iran.*; c *Department of Food Science and Technology, National Nutrition and Food Technology Research Institute, Faculty of Nutrition Sciences, Food Science and Technology, Shahid Beheshti University of Medical Sciences, P.O. Box 19395-4741, Tehran, Iran. *; d *Department of Food Technology Research, National Nutrition and Food Technology Research Institute, Shahid Beheshti University of Medical Sciences, P.O. Box 19395-4741, Tehran, Iran. *

**Keywords:** Doogh, Inoculation, Probiotic, Texture

## Abstract

Effects of inoculation level (4 or 8-fold compared to standard inoculation) and order (standard inoculation before fermentation and 3-fold inoculation at the end of fermentation = 1+3, Two-fold inoculation before fermentation and the same at the end of fermentation = 2+2, 2+6, 4-fold before fermentation = 4, 4+4, and 8) of culture inoculums containing probiotics on viscosity, phase separation, particle size analysis, microstructure and sensory attributes of probiotic Doogh were studied. The probiotic microorganisms were *Lactobacillus acidophilus *LA-5 and *Bifidobacterium lactis *BB-12. Treatments with 2- and 4-fold inoculation before fermentation had the highest instrumental viscosity and surface tension at the end of fermentation. The size diameter of particles in the structure of treatment 8I was significantly (p < 0.05) smaller than I after stirring with a Lab stirrer (1500 rpm), and even after homogenization with a homogenizer (150 bar). 8I was an un-uniform, disintegrated and clumped structure with limited junctions in its network that resulted in a weak structure with bigger particles after agitation and smaller particles after stirring and homogenization compared to other treatments. This treatment also had the lowest record in ranking sensory test among treatments with a mixed culture-like and vinegary-like taint. Overall, treatments with 2- and 4-fold inoculation were realized as the best from the sum of physiochemical and sensory properties point of view.

## Introduction

‘Functional foods’ are foods that are not consumed only to satisfy basic nutritional demands, but also to exhibit extra health properties to the consumers (1). Many people are willing to continuously consume functional foods to prevent different diseases instead of incorporating drugs and supplements and this fact indicates the special place and popularity of functional foods in public health (2, 3). One of the most promising approach to extend the area of functional foods in food industry is via ‘probiotic’ technology, especially in dairy industry (2). Probiotics are particular species and strains of microorganisms that imply health promotions to the consumers provided be ingested to sufficient amounts continuously (3). Therefore, this important parameter that is known as ‘viability’ (the minimum viable probiotic cells for each strain in g or mL of product until the time of consumption) in food industry is the most critical value of probiotic products. Although no global and unique standard is exist regarding the viability of probiotics in different products, generally, the level of 10^7^ cfu/mL has been accepted as a minimum viability in most of products (4).

Besides the viability of probiotics, it is important that incorporation of probiotic bacteria should not affect the expected sensory characteristics (flavor, texture, and appearance) of conventional product. However, their addition might contribute to weak sensory attributes due to non-extended flavor and texture and/or emergence of taint (5). Therefore, it is important to compare probiotic food products with non-probiotic controls through sensory evaluation when developing new products. It is known that sensory characteristics of functional food products are superior to their health considerations for consumers (4). In other words, consumers are not interested in consuming a functional food even with valuable health benefits with disagreeable sensory properties. In this research, following to related previous research about considering the effects of these factors on microbiological and biochemical characteristics of probiotic Doogh (6), the effects of inoculation level and order (before or before plus after) of culture inoculum in textural, structural and sensory characteristics of probiotic Doogh were investigated. 

## Experimental

*Probiotic bacteria*

Lyophilized culture that commercially known as ABY-type including yogurt bacteria (mixed culture of *Streptococcus thermophilus *and *Lactobacillus delbrueckii *ssp. *bulgaricus*), *L. acidophilus *LA-5 and *Bifidobacterium lactis *BB-12 were supplied by Chr-Hansen (Horsholm, Denmark). The cultures were maintained according to manufacturer’s instructions at -18°C, until used.

*Sample preparation and study design*

Doogh with 4% of solid non-fat milk was made by reconstitution of skim milk powder (Pak Co., Tehran, Iran). Then, the mixture was incorporated with 0.7% (m/m) industrial sodium chloride. The milk was heat treated (90°C/15 min) and after cooling down to fermentation temperature (40*°*C), samples with primary fermentation were inoculated with ABY-type culture in different states: according to the manufacturer’s instruction as standard inoculation (I), 2-fold of ‘I’ (2I), 4-fold of ‘I’ (4I), or 8-fold of ‘I’ (8I). Fermentation was carried out until a pH of 4.2 *± 0.02 was reached. *At the end of fermentation, for treatments with sequential inoculation, the secondary inoculations were carried out: 3-fold following previous 1-fold = (1 + 3)I, 2-fold following the previous 2-fold = (2 + 2)I, 6-fold following the previous 2-fold = (2 + 6)I, and 4-fold following the previous 4-fold = (4 + 4)I. Then, all treatments in PET bottles were shacked uniformly and subjected to experiments. Viscosity change of treatments during fermentation and viscosity and surface tension of treatments at the end of fermentation, phase separation during 20 days of refrigerated storage (4°C), particle size analysis at the end of fermentation, and sensory properties were assessed. No essence was added in order to discriminate any probable fine differences among treatments in sensory testing.

*Viscosity and surface tension analysis*

Viscosity of Doogh samples was assessed using Brookfield rotational viscometer (Brookfield, USA) in four rotational velocities including 10, 20, 50 and 100 rpm. Surface tension of the samples was measured using tensiometer (Kruss, Germany). Determinations were done at 20ºC (7).

*Particle size distribution analysis*

The particle size distribution was analysed using the light scattering method and Mastersizer instrument (MAL 101594, Malvern, UK). The assessed indexes were minimum diameter (d_m_- μm), maximum diameter (d_M_-μm), peak diameter (d_p_-μm; the highest percentage in diameter distribution of particles), range of diameter (d_R_-μm; d_M_-dm), diameters of 10, 50 and 90% (d_10_, d_50_ and d_90_-μm; diameters that 10%, 50% and 90% of particle sizes are below them), span (d_90_-d_10_/d_50_) and specific surface area (the surface area of mass unit of particles: m^2^/g) (8).

*Light microscopy*

0.5 mL of rhodamin B solution (0.01% m/m) (Merck, Darmstadt, Germany) was added into 10 mL test portion of Doogh for staining the casein particles and after stirring, a droplet was applied for direct light microscopic observation and preparing microscopic images using phase contrast state (microscope: E1000, Nikon, Japan; digital camera: DXM-1200, Nikon, Japan). The magnification was ×400 (9).

*Phase separation analysis*

Doogh samples were equally poured in similar test tubes (closed cap) and stored in refrigeration temperature (5ºC) in a still state. During 20 days of storage, the height of upper phase (transparent phase or supernatant) was measured and the percentage of phase separation was calculated as using following equation (9):

Phase separation (%) = height of supernatant/total height of sample in text tube ×100 

*Sensory analysis*

A trained consumer panel of 9 panelists made the sensory analysis. The treatments were compared using ranking test. The panelists were asked to rank the treatments in order from quality parameters points of view. The quality parameters were sourness, off-flavor, texture smoothness, oral viscosity, texture smoothness, mouthfeel, stringiness, saltiness, opacity, aroma intensity and overall acceptance (7). 

*Statistical analysis*

All results were an average of three replicate determinations and the significant differences (p < 0.05) among the means were analyzed using the one-way and two-way ANOVA test (based on the complete randomized design-full Factorial test design) from Minitab software (Version 13, 2002).

## Results and Discussion

*Viscosity, surface tension, particle size analysis and microstructure of treatments*

Table 1 shows viscosity change (cp) in Doogh milk of different treatments during fermentation. 

**Table 1:** Viscosity change (cp) in Doogh milk of different treatments during fermentation in four rotational velocities of viscometer*

Treatment	Fermentation time (min) and four rotational velocities of viscometer (rpm)											
	0 (N)**	120 (N)	240 min (nN)					270 or 330 or 360 min (nN)****				
			10 (rpm)	20 (rpm)	50 (rpm)	100 (rpm)	Trend (cp)	10 (rpm)	20 (rpm)	50 (rpm)	100 (rpm)	Trend (cp)
I/(1+3)I ***	7.0^a^	7.5^c^	40.0^b^	30.0^a^	24.5^a^	35.0^a^	-10/-5.5/+10.5	62.5^b^	40.0^b^	26.9^b^	29.0^b^	-12.5/-13.1/+2.1
(2+2)I/(2+6)I	7.1^a^	10.75^b^	40.0^b^	25.0^b^	19.0^b^	25.5^c^	-15/-6/+6.5	75.0^a^	50.0^a^	32.0^a^	34.0^a^	-25/-18/+2
4I/(4+4)I	7.2^a^	10.5^b^	45.0^a^	30.0^a^	25.0^a^	37.5^a^	-10/-5/12.5	63.5^b^	41.1^b^	26.9^b^	34.3^a^	-22.4/-14.2/+7.4
8I	7.3^a^	15.6^a^	41.0^b^	25.1^b^	20.0^b^	31.2^b^	-15.9/-5.1/+11.2	40.0^c^	30.0^c^	23.0^c^	30.0^b^	-10/-7/+7

*Means in the same column shown with different letters are significantly different (*p*<0.05).

** N = Newtonian, nN = non-Newtonian

*** I = standard inoculation, 4I = 4-fold inoculation, 8I = 8-fold inoculation, (1+3)I = standard inoculation before fermentation (primary inoculation) and 3-fild inoculation at the end of fermentation (secondary inoculation), and etc.

**** Fermentation time was 360 for I/(1+3)I, (2+2)I and (2+6)I; 330 for 4I and (4+4)I; and 270 for 8I.

Table 2 implies viscosity and surface tension of treatments at the end of fermentation. As appeared, after 120 min of fermentation, viscosity was in direct correlation with the inoculum level before fermentation and the treatment 8I had the greatest Newtonian viscosity. 

**Table 2:** Viscosity (cp; at different rotational velocities of viscometer-rpm) and surface tension (mN/m^2^) of treatments at the end of fermentation in four rotational velocities of viscometer*

Treatment	Viscosity						Surface tension (mN/m^2^)
	Newtonian (cp)	Non-Newtonian (cp)					
		10 (rpm)	20 (rpm)	50 (rpm)	100 (rpm)	Trend (cp)	
[I/(1+3)I]-S**	-	28.0^ab^	18.0^ab^	16.1^a^	18.0^ab^	-10/-1.9/+1.9	44.8^a^
[I/(1+3)I]-H	11.8^b^	-	-	-	-	-	40.7^bc^
[(2+2)I/(2+6)I]-S	-	30.0^a^	20.0^a^	17.0^a^	20.5^a^	-10/-3/+3.5	45.0^a^
[(2+2)I/(2+6)I]-H	14.7^a^	-	-	-	-	-	42.7^ab^
[4I/(4+4)I]-S	-	30.0^a^	21.0^a^	17.8^a^	21.0^a^	-9/-3.2/+3.2	45.2^a^
[4I/(4+4)I]-H	12.4^ab^	-	-	-	-	-	42.4^ab^
8I-S	14.5^a^	-	-	-	-	-	41.8^b^
8I-H	11.9^b^	-	-	-	-	-	41.4^b^

*Means in the same column shown with different letters are significantly different (*p*<0.05).

** I = standard inoculation, 4I = 4-fold inoculation, 8I = 8-fold inoculation, (1+3)I = standard inoculation before fermentation (primary inoculation) and 3-fild inoculation at the end of fermentation (secondary inoculation), and etc./ -S = stirred before experiment; -H = homogenized with 120 bar pressure before experiment

The lowest viscosity belonged to the treatments with standard inoculation before fermentation. The reason could be on one hand, faster acidification in treatments with higher initial levels of inoculation that led to the sooner formation of protein aggregates and on the other hand, faster formation of exo-cellular polysaccharides (EPSs) produced by the starter bacteria due to their greater growth and activity (9). However, from that time on, the viscosity increased in treatments with higher initial inoculation levels (8- and 4-fold) and continued slower compared to those with smaller inoculation level (2-fold and I) in such as way that at final stages of fermentation, treatment with 2-fold initial inoculation (2 + 2 or 2 + 6) possessed the highest viscosities in all rotational velocities of viscometer (10, 20, 50 or 100). Therefore, after a definite time of fermentation, initial inoculation level showed reverse correlation with the amounts of viscosity. This observation can be justified as follow: in treatments with high inoculation levels (4- or 8-fold), at the time range of 150-240 during fermentation, the starter cultures were in stationary phase of growth (data no shown), in which the EPSs were being widely produced by them. Excessive amounts of produced EPSs (especially within the time range of 120-150, when the protein aggregates are not formed enough toward a gel structure) might prevent adequate fusion of casein micelles and prevent formation of a continuous and integrated three-dimensional gel network structure (10) and therefore, the viscosity increase rate was reduced during fermentation. 

**Figure 1:**
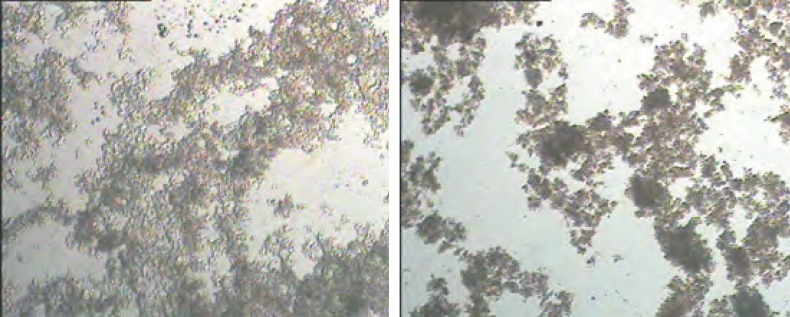
Light microscopic images of I (a) and 8I (b) treatments. The samples were not homogenized with homogenizer and were only uniformly agitated before analysis

Another mechanism to justify aforementioned observation could be clumped aggregation phenomenon in treatments with higher inoculation levels. These treatments exhibit faster acidification rates during fermentation. Faster acidification leads to faster aggregation of casein micelles and particulate due to shorter available time that leads to formation of un-uniform and disintegrated aggregates that are far from a three-dimensional gel network structure. Such structures consist of large aggregates and mentioned mechanism is called ‘clumped aggregation’. Visa versa, slower acidification during fermentation leads to formation of uniform and continuous structure with many junctions points in gel network that is known as ‘linear aggregation’ (11, 12). The former structure results in dispersions with greater particle size after gel disruption and stirring. Figure 1 represents light microscopic images of treatments I and 8I. As indicated, the microstructure of treatment ‘I’ consists of a continuous-integrated structure with universal junctions in contrast to 8I with clumped structure made of limited junctions. Such structures (I) possessed significantly (p < 0.05) higher resistance against shear and flow forces that leads to higher viscosity. 

Table 4 represents Particle size analysis parameters for treatments at the end of fermentation. As appeared, the size diameter of particles in the structure of treatment 8I was significantly (p < 0.05) smaller than I after stirring with a Lab stirrer (1500 rpm), and even after homogenization with a homogenizer (150 bar). This indicates less junctions and interactions in the structure of 8I compared to ‘I’ that leads to more particlized and dispersed structure after vigorous agitation and homogenization as well as to lower viscosity. The interesting point was that in 8I, after stirring (not homogenization), the viscosity was completely Newtonian, indicating an initial disintegrated structure. According to Table 2, treatments with 2- and 4-fold inoculation before fermentation had the highest viscosity and surface tension at the end of fermentation in all rotational velocities of viscometer. It seems that this inoculation level on one hand was not high enough to make a weak structure like as that in 8I and on the other hand, the starter cells were in population to make adequate (not less and not more) EPSs to strengthen the gel structure. According to mentioned Table, all treatments showed rheopectic behavior from the second rotational velocity to the third (50-100). 

*Phase separation during storage time*

Table 3 shows phase separation percent in different treatments during 21 days of refrigerated storage (4ºC). No considerable difference was observed between stirred or homogenized treatments in phase separation. This means that vigorous stirring or homogenizing the samples (the second is normally occurring in industry) made the particle characteristics of treatments near to each other. Therefore, protein systems in treatments did not show different separation behavior from serum phase. According to this Table, it appeared that homogenized trials render significantly (p < 0.05) higher phase separation within the 5 days of storage than stirred ones with a lower increase in mentioned parameter afterwards. It is clear and reasonable that the dispersed systems with smaller particle size display greater density that make them more capable of separation from serum phase (9).

**Table 3:** Phase separation (%) in different treatments during 21 days of refrigerated storage (4ºC).*

Treatment	Storage time (d)				
	5	10	15	20	Trend (%)
I-S**	48.6^cdD^	54.6^cdC^	59.4^cAB^	61.4^cA^	11.1/9.9/3.2
I-H	67.7^abC^	71.1^abB^	73.8^aA^	74.2^abA^	5/3.8/5
(1+3)I-S	49.6^cB^	57.2^cA^	57.8^cA^	57.8^cA^	15.3/1/0
(1+3)I-H	72.8^aC^	75.1^aB^	76.4^aAB^	77.1^aA^	3.2/1.8/0.9
(2+6)I-S	52.1^cB^	54.3^cdAB^	55.9^cdA^	56.3^cdA^	4.2/2.9/7
(2+6)I-H	68.8^abB^	70.4^abAB^	71.8^abA^	71.8^abA^	2.3/1.9/0
(2+2)I-S	44.8^dC^	51.7^dB^	53.8^cdAB^	55.7^cdA^	15.4/4.1/3.5
(2+2)I-H	60.7^bC^	68.3^bAB^	69.6^bA^	70.3^bA^	12.5/1.9/1
(4+4)I-S	51.1^cB^	53.2^cdAB^	54.7^cdA^	55.1^cdA^	4.1/2.8/0.7
(4+5)I-H	67.2^abB^	69.0^bAB^	70.1^abA^	70.6^bA^	2.7/1.6/0.7
4I-S	50.0^cD^	55.9^cdC^	58.0^cAB^	59.4^cA^	11.8/3.7/2.4
4I-H	71.4^aC^	74.5^aAB^	75.3^aA^	75.6^abA^	4.3/1.1/0.4
8I-S	51.4^cB^	59.3^cA^	59.3^cA^	59.3^cA^	15.3/0/0
8I-H	62.5^bC^	71.2^abB^	73.1^abAB^	74.0^abA^	14.1/2.7/1.2

*Means shown with different small and capital letters represent significant differences (*p *<0.05) in the same columns and rows, respectively.

** I = standard inoculation, 4I = 4-fold inoculation, 8I = 8-fold inoculation, (1+3)I = standard inoculation before fermentation (primary inoculation) and 3-fild inoculation at the end of fermentation (secondary inoculation), and etc./ -S = stirred before experiment; -H = homogenized with 150 bar pressure before analysis.

**Table 4:** Particle size analysis parameters for treatments at the end of fermentation (before refrigerated storage).*

		Parameters							
Treatment	d_m_*** (μm)	d_p _(μm)	d_M _(μm)	d_R _(μm)	span	d_10 _(μm)	d_50 _(μm)	d_90 _(μm)	SSA (m^2^/g)
[I/(1+3)I]-S**	0.48^a^	32.44^a^	416.78^a^	384.43^a^	1.91^bc^	7.41^a^	26.10^a^	57.13^a^	0.46^cd^
[I/(1+3)I]-H	0.32^b^	6.18^e^	104.71^b^	103.61^b^	2.37^a^	1.77^d^	5.80^d^	15.50^e^	1.62^b^
[(2+2)I/(2+6)I]-S	0.48^a^	24.61_b_	91.20^c^	90.10^c^	1.74^d^	6.76^ab^	22.43^ab^	45.84^b^	0.51^cd^
[(2+2)I/(2+6)I]-H	0.32^b^	5.38^ef^	69.18^d^	68.08^d^	1.97^b^	1.53^de^	4.86^d^	11.14^ef^	1.89^ab^
[4I/(4+4)I]-S	0.28^bc^	18.67^c^	69.18^d^	68.10^d^	1.74^d^	4.64^c^	15.53^c^	31.61^c^	0.73^c^
[4I/(4+4)I]-H	0.28^bc^	5.38^ef^	19.95^f^	18.85^f^	1.72^d^	1.49^e^	4.48^d^	9.19^f^	2.03^a^
8I-S	0.28^bc^	16.26^cd^	52.48^e^	51.38^e^	1.70^de^	4.48^c^	14.29^c^	28.71^cd^	0.77^c^
8I-H	0.24^c^	4.69^f^	19.95^f^	18.85^f^	1.73^d^	1.44^e^	4.09^d^	8.52^fg^	2.17^a^

*Means in the same column shown with different letters are significantly different (*p*<0.05).

** I = standard inoculation, 4I = 4-fold inoculation, 8I = 8-fold inoculation, (1+3)I = standard inoculation before fermentation (primary inoculation) and 3-fild inoculation at the end of fermentation (secondary inoculation), and etc./ -S = stirred before experiment; -H = homogenized with 120 bar pressure before experiment

*** d_m_ = minimum diameter, maximum diameter (-μm), peak diameter (d_p_-μm; the highest percentage in diameter distribution of particles), range of diameter, d_M _= maximum diameter, d_p_ = peak diameter, d_R_ = range of diameter (d_M_-d_m_), d_10_, d_50_ and d_90 _= diameters of 10, 50 and 90% diameters that 10%, 50% and 90% of particle sizes are below them), span = d_90_-d_10_/d_50 _and SSA = specific surface area (the surface area of mass unit of particles).

*Sensory analysis*

Table 5 indicates ranking sensory test among treatments at the end of fermentation. Following results could be obtained:

a) Very high inoculation (8I) led to a specific combined off-flavor that was known as ‘culture-like’ and ‘vinegary-like’ taints. The second must have been probably generated by the over-population of bifidobacteria. This noticeable off-flavor caused the treatment 8I had the lowest overall acceptance among treatments. b) Oral viscosity was the highest in 4I and the lowest in 8I. This observation is in line with previous results regarding instrumental viscosity. c) Treatments with 2- and 4-fold inoculation before fermentation had the best mouthfeel and texture smoothness. This could be attributed to the adequate amounts of produced EPSs by starter culture. It has been reported that EPSs in adequate amounts can significantly (p < 0.05) improve mouthfeel and texture smoothness (10). The lowest grade belonged to the 8I, their samples occasionally had the defect of nodulation and clumping (the samples were not stirred or homogenized), indicating un-uniform structure that appeared in clumped particles after agitating. 

d) The treatment 4I rendered the highest stringiness which represents presence of adequate amounts of EPSs produced by starter cultures during fermentation. EPSs can increase the extensibility of liquids during pouring (10). e) The treatment 4I possessed the sense of being more salty. This characteristic could be attributed to enhancing effect of EPSs on saltiness perception. The reason that why 8I (which contained more amounts of EPSs) was not perceived saltier than 4I could be attributed to the masking effect of off-flavors in latter trial. Also, EPSs probably renders masking impact on aroma perception because the highest aroma intensity was reported in I and then 2I rather than 4I and 8I. In 8I, the emerged taint also reduced aroma intensity more than other treatments. 

**Table 5:** Ranking test among treatments at the end of fermentation (*p*<0.05)*

Treatment	Sensory parameters								
	Sourness	Off-flavor	Oral viscosity	Texture smoothness	Mouthfeel	Stringiness	Saltiness	Aroma intensity	Total acceptance
I, 2I, 4I, 8I	NS***	8I>4I (a mixed culture-like and vinegary-like off-flavor)	4I>2I>I>8I	Others>8I	4I=2I>others	4I>others	4I>2I=8I>I	I>2I>4I>8I	Others>8I

* All the treatments were uniformly agitated in bottles before sensory tests and did not stirred or homogenized.

** I = standard inoculation and coefficients to ‘I’ are inoculations more than standard inoculation before

*** Non-significant

## Conclusion

This work demonstrated that inoculation level and sequence significantly (p < 0.05) affected the physicochemical and sensory attributes of treatments. Treatments with 2- and 4-fold inoculation before fermentation had the highest instrumental viscosity and surface tension at the end of fermentation. 8I was an un-uniform, clumped and weak structure compared to other treatments especially ‘I’. This trial also showed the lowest sensory acceptance with a mixed culture-like and vinegary-like taint. Overall, treatments with 2- and 4-fold inoculation were realized as the best from the sum of physiochemical and sensory properties point of view. 
